# Supplementary material for: Prognostic Value of the Diversity of Nuclear Chromatin Compartments in Gynaecological Carcinomas
Source: Cancers (Basel). 2020 Dec 19;12(12):3838. doi: 10.3390/cancers12123838 (PMC7766595; doi:10.3390/cancers12123838)
Supplement: Supplementary file 1 [file cancers-12-03838-s001.pdf]

Supplementary Materials

To the article by A. Kleppe, F. Albregtsen, J. Trovik, G. B. Kristensen and H. E. Danielsen, titled: Prognostic Value of the Diversity of Nuclear Chromatin Compartments in Gynaecological Carcinomas

S1 Segmentation of Chromatin Compartments

An algorithm for segmentation of visible chromatin compartments in nuclear images was developed using the train subset of the ovarian carcinoma cohort. An important challenge implied by acquiring two-dimensional images of entire cell nuclei, is that different chromatin compartments may overlap in a nuclear image. Also, the physical size in the direction of projection may differ across an imaged nucleus, typically being thinner near the periphery than the centre, warranting the segmentation approach to be locally adaptive.

Technically, the segmentation was performed by assigned one of three unique labels to each pixel in a nuclear image. Two of the labels were used to indicate pixels in relatively highly condensed or relatively weakly condensed chromatin compartments, while the last label was used to indicate that the pixel was not a part of such compartments. The appropriate label for each pixel was automatically determined using two segmentation steps. The first step adaptively thresholded the nuclear image, and the second separated overlapping chromatin compartments and removed small objects. These steps are described in each of the two following subsections.

S1.1 Adaptive Thresholding

A nuclear image $M \in \{0, 1, \dots, G-1\}^{m,n}$, where G is the number of grey levels and m and n are the height and width of the image, was thresholded using a generalisation of Niblack's adaptive thresholding algorithm [1]:

1. Calculate the gradient magnitude image using the 3x3 Sobel operator.

2. For every $s = (w, k_d, k_b)$ tuple with window size $w \in \{5, 7, 9\}$ and threshold parameters $k_d, k_b \in \{0.3, 0.4, \dots, 1.0\}$, compute the thresholded image $N_s \in \{0, 1, 2\}^{m,n}$ by:

$$N_s(i,j) = \begin{cases} 0 & \text{if } M(i,j) < t_d(i,j) \\ 1 & \text{if } t_d(i,j) \leq M(i,j) \leq t_b(i,j) \\ 2 & \text{if } M(i,j) > t_b(i,j) \end{cases} \tag{1}$$

where 0, 1 and 2 indicates dark, grey and bright region, respectively, representing chromatin compartments with relatively high, intermediate and weak condensation, and:

$$t_d(i,j) := \mu_w(i,j) - k_d\sigma_w(i,j) \tag{2}$$

$$t_b(i,j) := \mu_w(i,j) + k_b\sigma_w(i,j) \tag{3}$$

for $i = 0, 1, \ldots, m-1$ and $j = 0, 1, \ldots, n-1$, and where $\mu_w(i,j)$ and $\sigma_w(i,j)$ were the expectation and standard deviation of the grey levels in a local window of size wxw with centre (i,j) in M.

3. The dark and bright criterion value for N_s was defined as the mean gradient magnitude of boundary pixels in dark and bright regions, respectively. The chosen thresholded image N was the N_s that maximised the mean of the dark and bright criterion values.

The chosen values of the threshold parameters k_d and k_b were distributed as shown in Supplementary Figure S4. The somewhat high lower bound of the threshold parameters, 0.3, assures that dark and bright regions are primarily separated by grey regions, a characteristic that was initially desired and later justified by evaluations in the train subset of the ovarian carcinoma cohort. These evaluations indicated that the classification accuracy was similar irrespective of whether the smallest possible value of the threshold parameters was 0.3, 0.2 or 0.1, and that accuracy was slightly lower if the smallest possible value was 0.

The window size chosen by the algorithm was 9 for all but one nuclear image. This was expected because an increased window size will shift the focus from highly local changes to larger trends, which in itself cause growing and joining of adjacent and prominent regions of the same type (dark or bright) and possibly shrinking or loss of smaller and more subtle regions, particularly if they are surrounded by both dark and bright regions. While the loss of subtle regions is likely to increase the criterion values, the growth of other objects may influence the criterion values either positively or negatively. However, if the criterion values were decreased, then the enlargement could be reversed by also increasing the threshold parameters, thus the combined result of increasing the window size and possibly the threshold parameters is fewer and more prominent objects, which should increase the criterion values. The choice of upper bound for the window size parameter is therefore essentially an assertion of the window size. Setting the value too high will often result in the selection of a thresholded image N where only the most prominent chromatin compartments are identified, but conversely, proper labelling of all relevant chromatin compartments requires that the desired compartments fit well within the windows. The applied upper bound, 9, is found to be a reasonable compromise.

S1.2 Splitting and Cleaning

As suggested by Yanowitz and Bruckstein [2] in the validation step of their segmentation method, the mean gradient magnitude of an object's boundary pixels reflects how clearly the object is distinguished from the background, making it a reasonable criterion for threshold fitness. However,

when multiple chromatin compartments of the same type (relatively highly condensed or relatively weakly condensed) overlap in a nuclear image, the gradient magnitude will not be high between the compartments, only between the cluster of compartments and the outside. Overlapping chromatin compartments can therefore not be expected to be separated in the thresholded image N, thus we need to detect and divide objects in N that encapsulate multiple chromatin compartments.

For each 8-connected dark or bright object in N, the corresponding sub-image in M was low-pass filtered by convolving it with:

$$\frac{1}{6}\begin{array}{|c|c|c|}\hline 0 & 1 & 0 \\ \hline 1 & 2 & 1 \\ \hline 0 & 1 & 0 \\ \hline\end{array}$$

The filtered image was watershed transformed and the label at the resulting 4-connected watershed lines was set as grey. Finally, dark or bright objects with less than five pixels were assigned the grey label.

The resulting image will subsequently be referred to as the *class label image* corresponding to the input nuclear image. Supplementary Figure S5 shows a representative nuclear image and the corresponding class label image. All prominent clusters of dark or bright pixels were detected as dark and bright objects, and the meaning of *cluster* depended on the local average intensity, as desired. Overlapping chromatin compartments seems appropriately handled; e.g., the relatively large dark cluster northwest of the centroid was separated into three reasonable dark objects.

S2 Dual Entropy Matrices

The *dual entropy matrix (DEM)* can be defined in the context described by Maître et al. [3] and Tupin et al. [4], where each pixel was characterised by the following three quantities:

- The optical density or grey level $g \in \{0, 1, \ldots, G_r - 1\}$ in a nuclear image uniformly requantified to $G_r = 64$ grey levels.
- The class label $l \in \{0, 1, \ldots, L-1\}$ in the corresponding class label image. $L = 3$ is the number of unique labels.
- The context value $v \in \{1, 2, \ldots, V\}$, here defined as the number of pixels in the 4-connected object in N that contains the pixel. V was the maximum object size.

Collecting the three values for all pixels in a nuclear images will give a grey level image $A \in \{0, 1, \ldots, G_r - 1\}^{m,n}$, a class label image $B \in \{0, 1, \ldots, L-1\}^{m,n}$ and a context value image $C \in \{1, 2, \ldots, V\}^{m,n}$.

Let $f(g, l, v)$ be the discrete probability that the combination of grey level g, class label l and context value v occurs in a specific (A, B, C)-tuple. By the definition of conditional probability and the law of total probability, the class conditional pmf is then:

$$f(g, v|l) = \frac{f(g, l, v)}{\sum_{g'=0}^{G_r-1} \sum_{v'=1}^{V} f(g', l, v')} \tag{4}$$

if the denominator is positive; otherwise, no pixels have class label l and we defined $f(g, v|l)$ as 0.

The marginals of the class conditional pmf are the grey level histogram for class l, $f(g|l)$, and the context value histogram for class l, $f(v|l)$:

$$f(g|l) := \sum_{v=1}^{V} f(g, v|l) = \frac{\sum_{v=1}^{V} f(g, l, v)}{\sum_{g'=0}^{G_r-1} \sum_{v=1}^{V} f(g', l, v)} \tag{5}$$

$$f(v|l) := \sum_{g=0}^{G_r-1} f(g, v|l) = \frac{\sum_{g=0}^{G_r-1} f(g, l, v)}{\sum_{g=0}^{G_r-1} \sum_{v'=1}^{V} f(g, l, v')} \tag{6}$$

Again the denominators are assumed positive, if not, then all $f(g, v|l)$ for class l are zero, which implies that the histograms $f(g|l)$ and $f(v|l)$ are also zero. Supplementary Figure S6 visualises the histograms for the bright label in the representative nuclear image.

The binary Shannon entropy of a grey level or context value histogram for class l was denoted ϵ_l and ζ_l, respectively, and defined as:

$$\epsilon_l := -\sum_{g=0}^{G_r-1} f(g|l) \log_2 f(g|l) \ , \ f(g|l) > 0 \tag{7}$$

$$\zeta_l := -\sum_{v=1}^{V} f(v|l) \log_2 f(v|l) \ , \ f(v|l) > 0 \tag{8}$$

We defined the DEM for class l and using q_{G_r} and q_V quantification levels per integer grey level and spatial entropy, respectively, as:

$$P := \delta(x - \lfloor q_{G_r} \epsilon_l \rfloor, y - \lfloor q_V \zeta_l \rfloor) \tag{9}$$

where δ is the Kronecker delta and the discrete variables x and y range from 0 to their respective theoretical maxima, which are the flooring of $q_{G_r} \log G_r$ and $q_V \log V$, respectively. The DEM of a nuclear image is thus a binary matrix where only element $(\lfloor q_{G_r} \epsilon_l \rfloor, \lfloor q_V \zeta_l \rfloor)$ has value one.

The DEMs of the nuclear images from a patient was averaged to obtain the patient's DEM, i.e. the element (x, y) of the kth patient's DEM for label l was:

$$\bar{P}_k(x, y|l) := \frac{1}{m_k} \sum_j P_{k,j}(x, y|l) \tag{10}$$

where $P_{k,j}$ was the DEM of the jth nuclear image from patient k, which had m_k nuclear images in total.

The DEM for the bright label is illustrated in Supplementary Figure S7 for a patient with good and a patient with poor clinical outcome. In this example, both entropies were typically larger for the patient with poor clinical outcome.

S3 Features from DEMs

Both predefined and adaptive features could be extracted from a patient's DEM. An adaptive feature method initiated by Walker et al. [5] and later developed by Albregtsen et al. [6–9] was

based on weighting each texture matrix element by its discriminatory ability. The feature value for label l would be a T-weighted sum of the patient's DEM, i.e.:

$$U_{k,l} := \sum_x \sum_y T(x,y|l)\bar{P}_k(x,y|l) \tag{11}$$

where the weight $T(x,y|l)$ should reflect the relative ability to discriminate between the two outcome classes in element (x,y) of the DEM for label l.

To compute the weight matrix T, consider the DEMs of the nuclear images from patients in a train subset. Let $P_{k,j}(x,y|l,\omega_c)$ be the value of element (x,y) in the DEM for label l of the jth nuclear image in the kth patient with outcome class ω_c. The class-specific arithmetic mean and unbiased sample variance at each element can then be written as:

$$\bar{P}(x,y|l,\omega_c) = \frac{1}{n_c}\sum_k \sum_j P_{k,j}(x,y|l,\omega_c) \tag{12}$$

$$S^2(x,y|l,\omega_c) = \frac{1}{n_c - 1}\sum_k \sum_j (P_{k,j}(x,y|l,\omega_c) - \bar{P}(x,y|l,\omega_c))^2 \tag{13}$$

where n_c is the number of DEMs in outcome class ω_c.

Walker et al. [5] suggested to measure the discriminatory ability in each element by the Mahalanobis distance between the outcome classes:

$$D(x,y|l) := \frac{|\bar{P}(x,y|l,\omega_0) - \bar{P}(x,y|l,\omega_1)|}{S(x,y|l)} \tag{14}$$

where ω_0 and ω_1 specifies the good and poor outcome class, respectively, and $S(x,y|l)$ is the square root of the unbiased pooled variance estimate:

$$S^2(x,y|l) := \frac{(n_0 - 1)S^2(x,y|l,\omega_0) + (n_1 - 1)S^2(x,y|l,\omega_1)}{n_0 + n_1 - 2} \tag{15}$$

The estimated Mahalanobis distances can be signed by the outcome class with the highest mean probability to form the weights, giving:

$$T(x,y|l) := \frac{\bar{P}(x,y|l,\omega_0) - \bar{P}(x,y|l,\omega_1)}{S(x,y|l)} \tag{16}$$

Recognise that $T(x,y|l)$ is the t-statistic in the two-sample t-test that assumes equal variances, under the null hypothesis that the expectations of the outcome classes are equal in element (x,y) for label l, when we disregard the constant scaling factor $(1/n_0 + 1/n_1)^{-1/2}$ that is irrelevant for our application.

Supplementary Figure S8 displays the weight matrix T for the dark and bright label when computed using the train subset of the ovarian carcinoma cohort. For both labels, poor clinical outcome was associated with higher entropies and the elements with high discriminatory ability formed two clusters. One of these clusters indicated good clinical outcome (depicted as bright

elements in Supplementary Figure S8) and the other indicated poor clinical outcome (depicted as dark elements in Supplementary Figure S8). Such clustering of highly discriminatory elements signifies consistent and large prognostic trends in the DEMs, and also reliable estimation of the discriminatory ability in the elements that form the clusters.

As the class-specific grey level entropy is likely to be positively correlated with the local grey level entropy, the increase in class-specific entropy associated with poor clinical outcome is in accordance with the prognostic shift in local entropy observed by Nielsen et al. [10]. The relatively high spatial entropy in the poor outcome class indicates that increased size and number of chromatin compartments is a characteristic of cancer cell nuclei in patients with worse clinical outcome. This finding in established cancer extends previous reports on the changes in size and number of chromatin compartments that occur during carcinogenesis [11–16].

S4 Dual Entropy Sum Histograms and Their Features

Each weight matrix shown in Supplementary Figure S8 strongly suggests that the same change in either entropy affects the discriminatory ability equally. If this holds in general, then all discriminatory information is maintained in the projection of the DEM onto its diagonal, i.e. in the sum histogram [17] of the DEM. We define the *dual entropy sum histogram (DESH)* as this projection:

$$\vec{p} := \delta(i - \lfloor q(\epsilon_l + \zeta_l) \rfloor) \tag{17}$$

where q is the number of quantification levels per integer entropy sum and the discrete variable i ranges from 0 to its theoretical maximum, which is the flooring of $q(\log G_r + \log V)$. An important advantage with performing the projection is that DESHs are more reliably estimated than DEMs due to much fewer elements, which also increases the accuracy of the computed weights in the adaptive feature method.

As for the DEMs, we could compute the signed Mahalanobis distance between good and poor clinical outcome at each DESH element. The resulting weight vectors in the train subset of the ovarian carcinoma cohort are illustrated in Supplementary Figure S9. As expected, the trends from the corresponding weight matrices persisted and the transitions became less noisy because of more reliable estimation.

It was far more common for a nuclear image to have entropy sums in the clusters of highly discriminatory elements than outside the clusters. Consequently, the estimation reliability varied across DESH elements, being highest in the clusters and relatively low outside of them. To obtain similar reliability in all elements, the element edges were automatically selected to give the same number of entropy sums in each element when considering only the nuclear images in the train subset. Evaluations in the train subset of the ovarian carcinoma cohort indicated that the best choice was three quantification levels that each contained the entropy sum of about 12000 nuclear images. When using the entire train subset, targeting to have the entropy sum of about 12000 nuclear images in each DESH element resulted in the weight vectors shown in Supplementary

Figure S10 and Supplementary Figure S11. These were used to compute adaptive feature values from DESHs in the same manner as described for DEMs.

Supplementary References

1. Niblack, W. *An Introduction to Digital Image Processing*, 2nd ed.; Prentice-Hall: Englewood Cliffs, NJ, USA, 1986; pp. 115–116.

2. Yanowitz, S.D.; Bruckstein, A.M. A New Method for Image Segmentation. *Comput. Vis. Graph. Image Process.* **1989**, *46*, 82–95, doi:10.1016/S0734-189X(89)80017-9.

3. Maître, H.; Bloch, I.; Sigelle, M. Spatial entropy: A tool for controlling contextual classification convergence. In Proceedings of the 1st International Conference on Image Processing, Austin, TX, USA, 13–16 November 1994; Volume 2, pp. 212–216, doi:10.1109/ICIP.1994.413562.

4. Tupin, F.; Sigelle, M.; Maître, H. Definition of a spatial entropy and its use for texture discrimination. In Proceedings of the 2000 International Conference on Image Processing, Vancouver, BC, Canada, 10–13 September 2000; Volume 1, pp. 725–728, doi:10.1109/ICIP.2000.901061.

5. Walker, R.F.; Jackway, P.T.; Longstaff, I.D. Recent Developments in the Use of the Co-occurrence Matrix for Texture Recognition. In Proceedings of the 13th International Conference on Digital Signal Processing, Santorini, Greece, 2–4 July 1997; Volume 1, pp. 63–65, doi:10.1109/ICDSP.1997.627968.

6. Albregtsen, F.; Nielsen, B.; Danielsen, H.E. Adaptive gray level run length features from class distance matrices. In Proceedings of the 15th International Conference on Pattern Recognition, Barcelona, Spain, 3–7 September 2000; Volume 3, pp. 738–741, doi:10.1109/ICPR.2000.903650.

7. Albregtsen, F.; Nielsen, B. Texture Classification based on Cooccurrence of Gray Level Run Length Matrices. *Aust. J. Intell. Inf. Process. Syst.* **2000**, *6*, 38–45.

8. Nielsen, B.; Albregtsen, F.; Danielsen, H.E. Low Dimensional Adaptive Texture Feature Vectors From Class Distance and Class Difference Matrices. *IEEE Trans. Med. Imaging* **2004**, *23*, 73–84, doi:10.1109/TMI.2003.819923.

9. Nielsen, B.; Albregtsen, F.; Danielsen, H.E. Statistical Nuclear Texture Analysis in Cancer Research: A Review of Methods and Applications. *Crit. Rev. Oncog.* **2008**, *14*, 89–164, doi:10.1615/CritRevOncog.v14.i2-3.10.

10. Nielsen, B.; Albregtsen, F.; Kildal, W.; Abeler, V.M.; Kristensen, G.B.; Danielsen, H.E. The prognostic value of adaptive nuclear texture features from patient gray level entropy matrices in early stage ovarian cancer. *Anal. Cell. Pathol.* **2012**, *35*, 305–314, doi:10.3233/ACP-2012-0065.

11. Boone, C.W.; Sanford, K.K.; Frost, J.K.; Mantel, N.; Gill, G.W.; Jones, G.M. Cytomorphologic evaluation of the neoplastic potential of 28 cell culture lines by a panel of diagnostic cytopathologists. *Int. J. Cancer* **1986**, *38*, 361–367, doi:10.1002/ijc.2910380310.

12. Danielsen, H.E. Premalignant Changes in DNA Organization in Mouse Liver After Diethylnitrosamine Treatment. Ph.D. Thesis, University of Oslo, Oslo, 1991; p. 40.

13. Deligdisch, L.; Miranda, C.; Barba, J.; Gil, J. Ovarian Dysplasia: Nuclear Texture Analysis. *Cancer* **1993**, *72*, 3253–3257, doi:c6fkdz.

14. Zink, D.; Fischer, A.H.; Nickerson, J.A. Nuclear structure in cancer cells. *Nat. Rev. Cancer* **2004**, *4*, 677–687, doi:10.1038/nrc1430.

15. Nandakumar, V.; Kelbauskas, L.; Johnson, R.; Meldrum, D. Quantitative Characterization of Preneoplastic Progression Using Single-Cell Computed Tomography and Three-Dimensional Karyometry. *Cytometry A* **2011**, *79A*, 25–34, doi:10.1002/cyto.a.20997.

16. Nandakumar, V.; Kelbauskas, L.; Hernandez, K.F.; Lintecum, K.M.; Senechal, P.; Bussey, K.J.; Davies, P.C.W.; Johnson, R.H.; Meldrum, D.R. Isotropic 3D Nuclear Morphometry of Normal, Fibrocystic and Malignant Breast Epithelial Cells Reveals New Structural Alterations. *PLoS One* **2012**, *7*, e29230, doi:10.1371/journal.pone.0029230.

17. Unser, M. Sum and Difference Histograms for Texture Classification. *IEEE Trans. Pattern Anal. Mach. Intell.* **1986**, *PAMI-8*, 118–125, doi:10.1109/TPAMI.1986.4767760.

18. Vergote, I.; Amant, F. Time to include high-risk early ovarian cancer in randomized phase III trials of advanced ovarian cancer. *Gynecol. Oncol.* **2006**, *102*, 415–417, doi:10.1016/j.ygyno.2006.08.001.

19. Njølstad, T.S.; Trovik, J.; Hveem, T.S.; Kjæreng, M.L.; Kildal, W.; Pradhan, M.; Marcickiewicz, J.; Tingulstad, S.; Staff, A.C.; Haugland, H.K.; et al. DNA ploidy in curettage specimens identifies high-risk patients and lymph node metastasis in endometrial cancer. *Br. J. Cancer* **2015**, *112*, 1656–1664, doi:10.1038/bjc.2015.123.

20. Hveem, T.S.; Njølstad, T.S.; Nielsen, B.; Syvertsen, R.A.; Nesheim, J.A.; Kjæreng, M.L.; Kildal, W.; Pradhan, M.; Marcickiewicz, J.; Tingulstad, S.; et al. Changes in Chromatin Structure in Curettage Specimens Identifies High-Risk Patients in Endometrial Cancer. *Cancer Epidemiol. Biomarkers Prev.* **2017**, *26*, 61–67, doi:10.1158/1055-9965.EPI-16-0215.

Supplementary Table S1. Characteristics of the 246 ovarian carcinoma patients

**Characteristic**	**Subgroup**	**Statistic**
Pathological risk classification* - no. (%)		
	Low risk	48 (20)
	Medium risk	67 (27)
	High risk	131 (53)
Diversity of chromatin compartments - no. (%)		
	Similar chromatin compartments	156 (63)
	Diverse chromatin compartments	90 (37)
Chromatin heterogeneity - no. (%)		
	Chromatin homogeneous	169 (69)
	Chromatin heterogeneous	77 (31)
GLEM4D classification† - no. (%)		
	Low chromatin entropy	164 (67)
	High chromatin entropy	82 (33)
Median age at surgery (IQR) - yr		57 (49-66)
Age at surgery - no. (%)		
	≤60 yr	141 (57)
	>60 yr	105 (43)
FIGO stage - no. (%)		
	IA	86 (35)
	IB	13 (5)
	IC	147 (60)
Histological grade - no. (%)		
	1	106 (43)
	2	36 (15)
	3	46 (19)
	Not graded (clear cell)	58 (24)
Histological type - no. (%)		
	Mucinous	65 (26)
	Endometrioid	49 (20)
	Serous	49 (20)
	Clear cell	58 (24)
	Small cell	2 (1)
	Mixed	8 (3)
	Unclassifiable	15 (6)
Dense adhesions - no. (%)		
	Absent	157 (65)
	Present	85 (35)
Rupture - no. (%)		
	Absent	128 (53)
	Present	115 (47)
Median follow-up time (IQR) - yr		11.7 (8.3-13.6)

FIGO, International Federation of Gynecology and Obstetrics; GLEM4D, four-dimensional grey level entropy matrix; IQR, interquartile range. *Pathological risk classification was: Low risk if FIGO stage IA and well differentiated; Medium risk if FIGO stage IA and well differentiated or FIGO stage IB or IC and moderately differentiated; High risk if either clear cell histology, poorly differentiated or both moderately differentiated and FIGO stage IB or IC [18]. †GLEM4D classification reported by Nielsen et al. [10].

Supplementary Table S2. Characteristics of the 791 endometrial carcinoma patients

Characteristic	Subgroup	Statistic
Pathological risk classification* – no. (%)		
	Low risk	610 (78)
	High risk	175 (22)
Diversity of chromatin compartments - no. (%)		
	Similar chromatin compartments	601 (76)
	Diverse chromatin compartments	190 (24)
Chromatin heterogeneity - no. (%)		
	Chromatin homogeneous	673 (85)
	Chromatin heterogeneous	118 (15)
GLEM4D classification† - no. (%)		
	Low chromatin entropy	573 (72)
	High chromatin entropy	218 (28)
Median age at primary treatment (IQR) - yr		66 (59-74)
Age at primary treatment - no. (%)		
	≤70 yr	507 (64)
	>70 yr	284 (36)
Primary treatment - no. (%)		
	Hysterectomy	767 (97)
	Tumour reduction	3 (0)
	Curettage	21 (3)
FIGO stage - no. (%)		
	IA	434 (55)
	IB	174 (22)
	II	55 (7)
	III	90 (12)
	IV	29 (4)
Pathologic node (N) stage - no. (%)		
	N0	517 (88)
	N1/2	72 (12)
Myometrial invasion - no. (%)		
	<50%	457 (65)
	≥50%	250 (35)
Histological grade - no. (%)		
	1	290 (37)
	2	265 (34)
	3	233 (30)
Histological type - no. (%)		
	Endometrioid carcinoma	665 (84)
	Serous carcinoma	53 (7)
	Clear cell carcinoma	30 (4)
	Carcinosarcoma	29 (4)
	Other	14 (2)
Median follow-up time (IQR) - yr		2.96 (1.47-4.44)

FIGO, International Federation of Gynecology and Obstetrics; GLEM4D, four-dimensional grey level entropy matrix; IQR, interquartile range. *Pathological risk classification was based on the curettage histology report and was: Low risk if benign, hyperplasia or endometrioid grade 1 or 2; High risk if non-endometrioid or endometrioid grade 3 [19]. †GLEM4D classification reported by Hveem et al. [20].

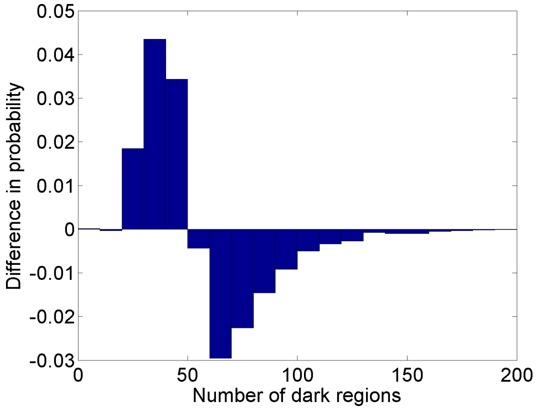

(a)

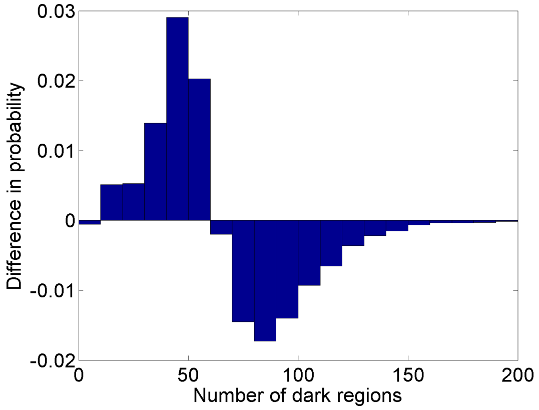

(b)

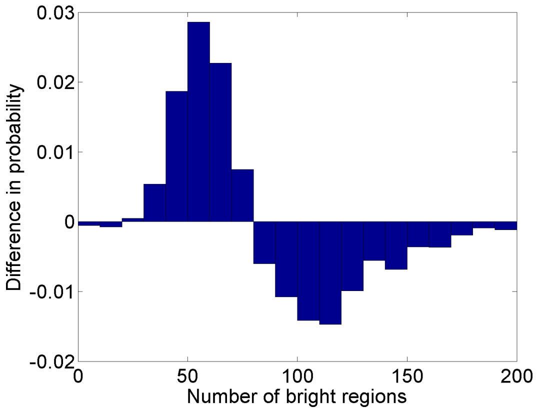

(c)

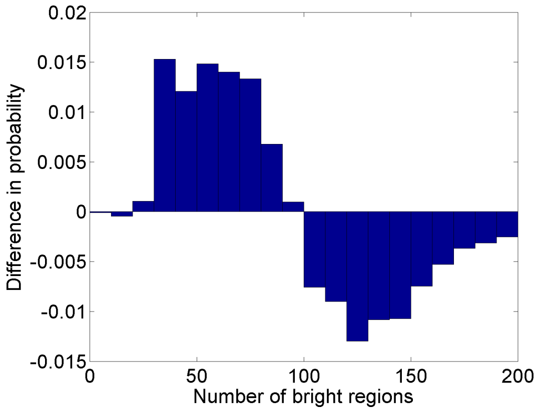

(d)

Supplementary Figure S1. Difference in distribution of number of regions from good to poor clinical outcome. (a) Dark regions in ovarian carcinoma cohort. (b) Dark regions in endometrial carcinoma cohort. (c) Bright regions in ovarian carcinoma cohort. (d) Bright regions in endometrial carcinoma cohort.

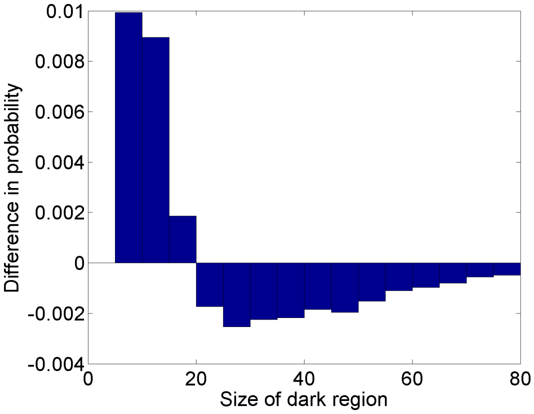

(a)

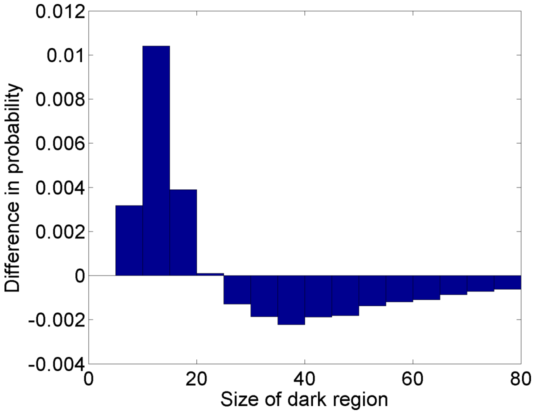

(b)

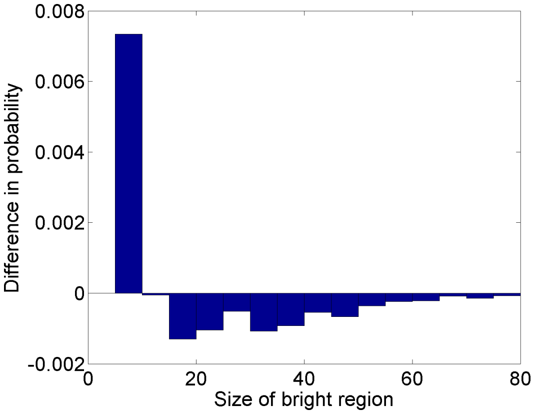

(c)

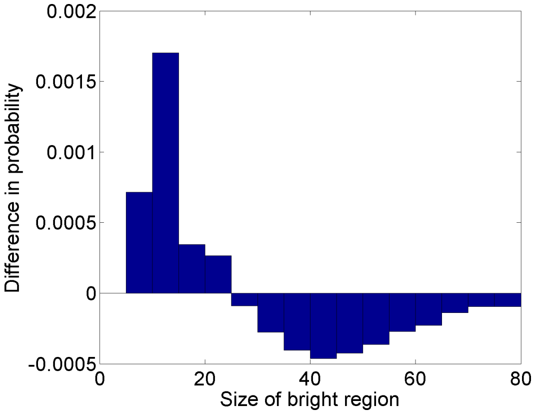

(d)

Supplementary Figure S2. Difference in distribution of chromatin compartment size from good to poor clinical outcome. (a) Dark regions in ovarian carcinoma cohort. (b) Dark regions in endometrial carcinoma cohort. (c) Bright regions in ovarian carcinoma cohort. (d) Bright regions in endometrial carcinoma cohort.

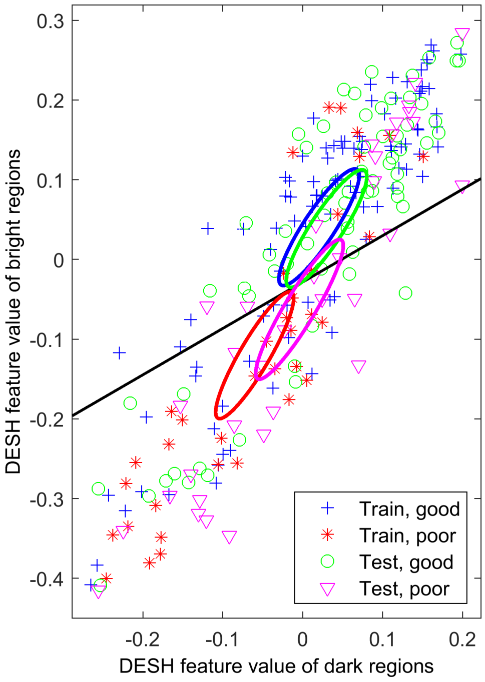

(a)

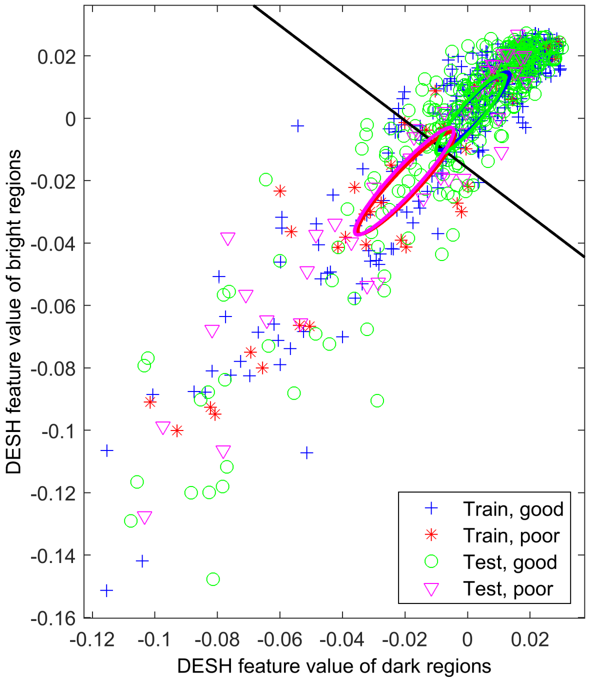

(b)

Supplementary Figure S3. Scatter plot of the dual entropy sum histogram (DESH) features in the (a) ovarian and (b) endometrial carcinoma patient cohort. The line in each scatter plot is the classifier's decision boundary when trained on the respective train subsets; the classification is diverse chromatin compartments (DCC) below the decision line and similar chromatin compartments (SCC) above the line. The ellipses are level curves at 0.2 of $(\vec{x}-\vec{m}_i)^T S_i^{-1}(\vec{x}-\vec{m}_i)$, where S_i is an unbiased feature sample covariance matrix and $\vec{m}_i$ is the corresponding mean feature vector.

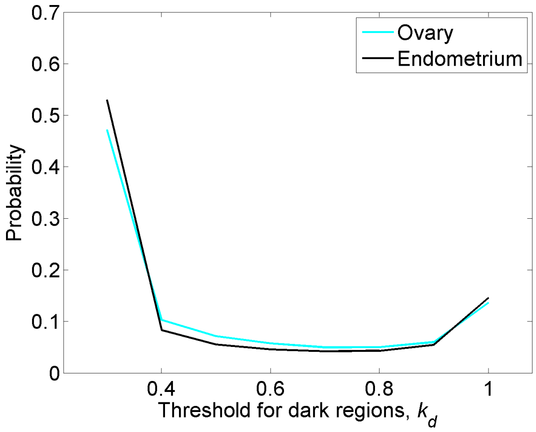

(a)

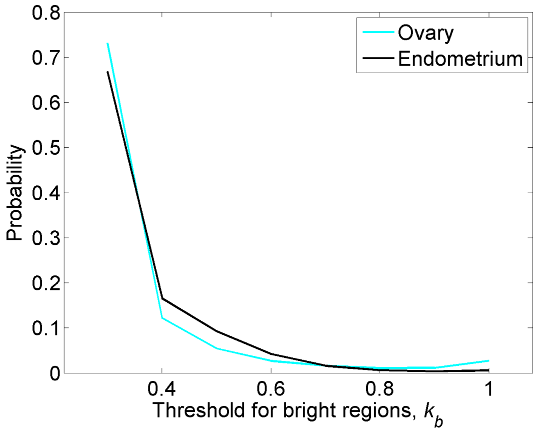

(b)

Supplementary Figure S4. Distribution of the chosen value of parameter (a) k_d and (b) k_b in the adaptive thresholding.

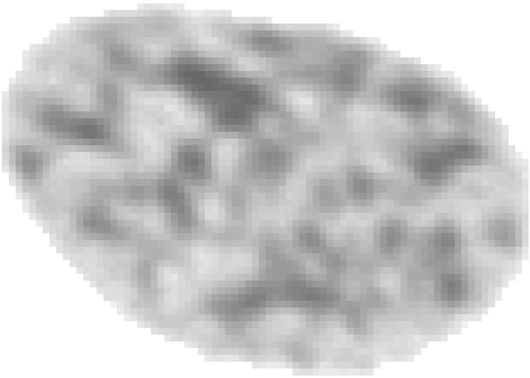

(a)

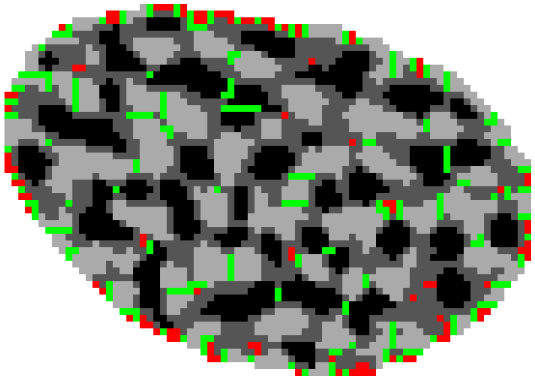

(b)

Supplementary Figure S5. (a) A representative nuclear image and (b) the corresponding class label image. The bright, grey and dark labels in the class label image are shown as light grey, dark grey and black, respectively. The applied watershed lines are shown in green, and the cleaned small objects are shown in red. The chosen parameters in the generalisation of Niblack's algorithm were $w = 9$, $k_d = 0.5$ and $k_b = 0.3$.

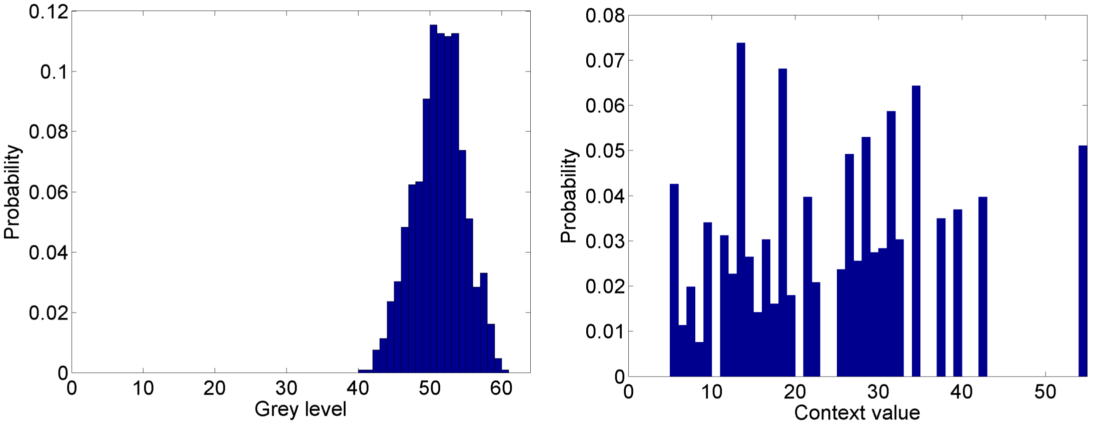

(a) (b)

Supplementary Figure S6. (a) Grey level and (b) context value histogram for the bright label of the nuclear image shown in Supplementary Figure S5.

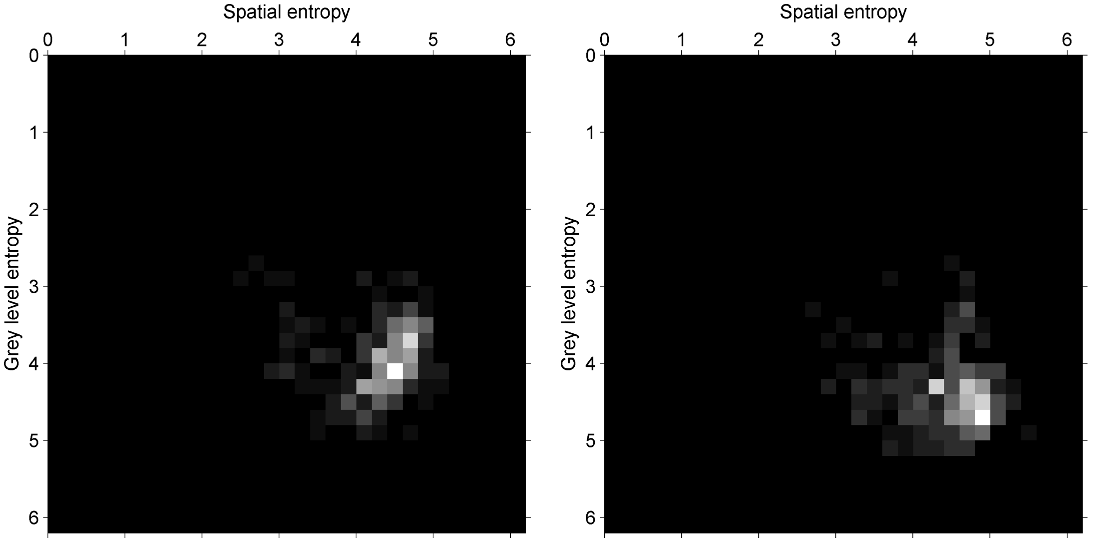

(a) (b)

Supplementary Figure S7. Dual entropy matrix (DEM) for the bright label of a ovarian carcinoma patient with (a) good clinical outcome and (b) poor clinical outcome when applying five quantification levels per integer entropy. The nuclear image in Supplementary Figure S5 is from the patient with good clinical outcome. The values of the DEMs range from 0 to 0.069 and 0.061, respectively.

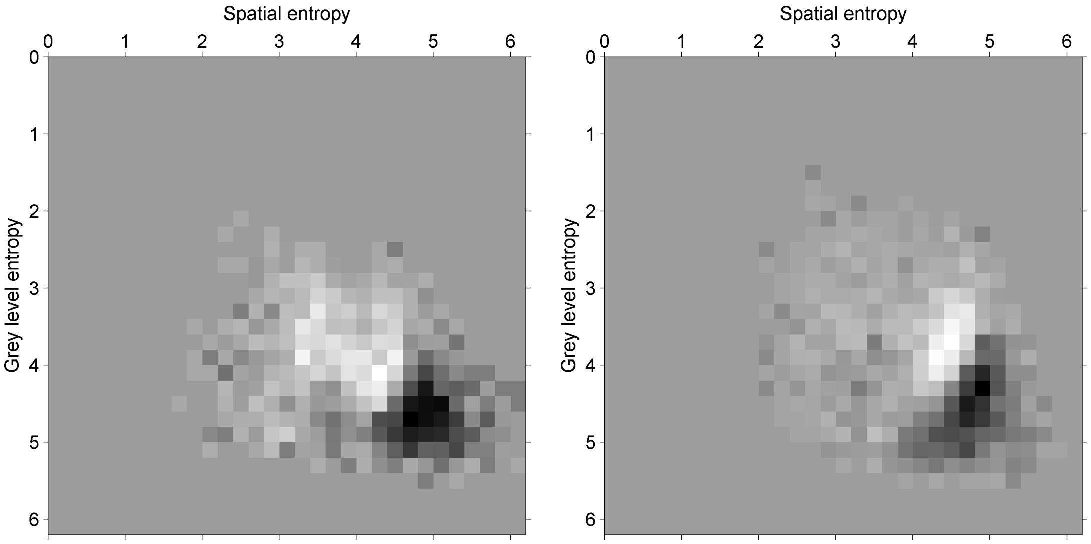

(a) (b)

Supplementary Figure S8. DEM weight matrix for the (a) dark and (b) bright label when applying five quantification levels per integer entropy. The weights were estimated using the nuclear images in the train subset of the ovarian carcinoma cohort and span $[-0.095, 0.060]$ and $[-0.154, 0.096]$, respectively.
DEM, dual entropy matrix.

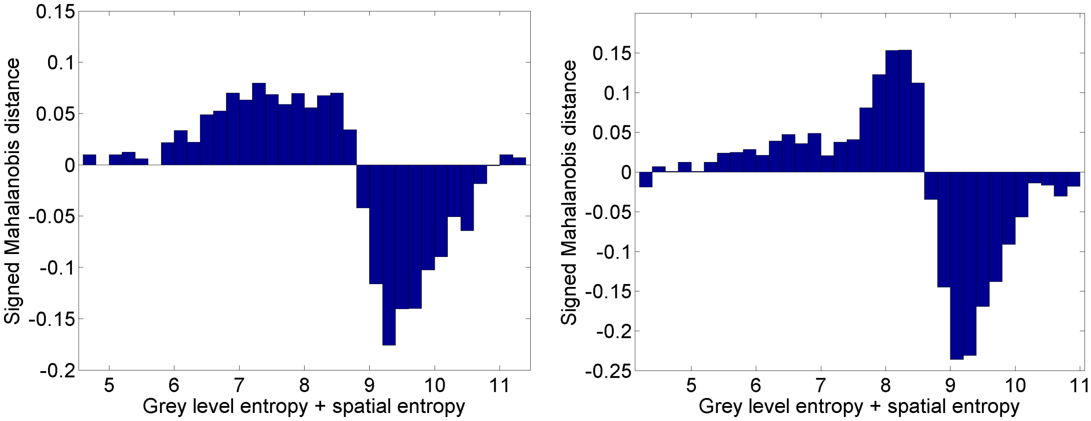

(a) (b)

Supplementary Figure S9. DESH weight vector for the (a) dark and (b) bright label when applying five quantification levels per integer entropy sum. The weights were estimated using the nuclear images in the train subset of the ovarian carcinoma cohort.
DESH, dual entropy sum histogram.

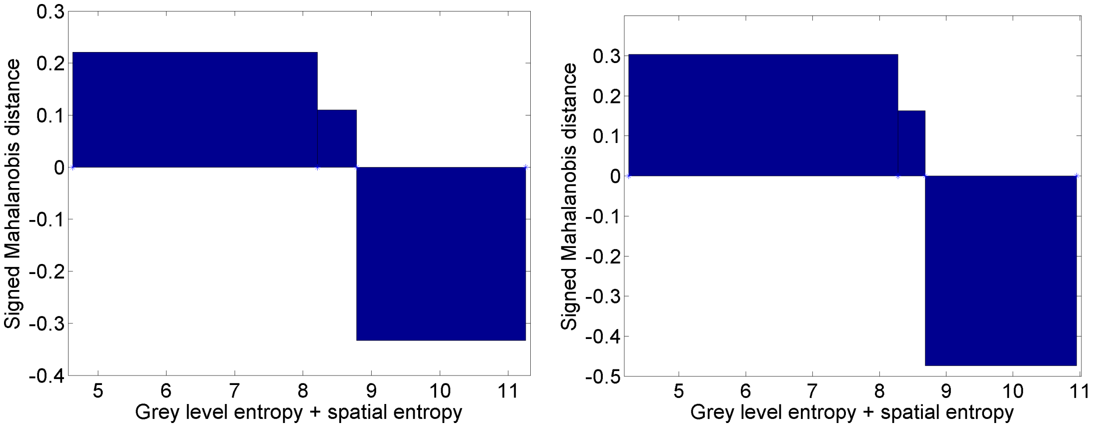

(a) **(b)**

Supplementary Figure S10. DESH weight vector for the (a) dark and (b) bright label when targeting 12000 entropy sums in each element. The weights were estimated using the nuclear images in the train subset of the ovarian carcinoma cohort.
DESH, dual entropy sum histogram.

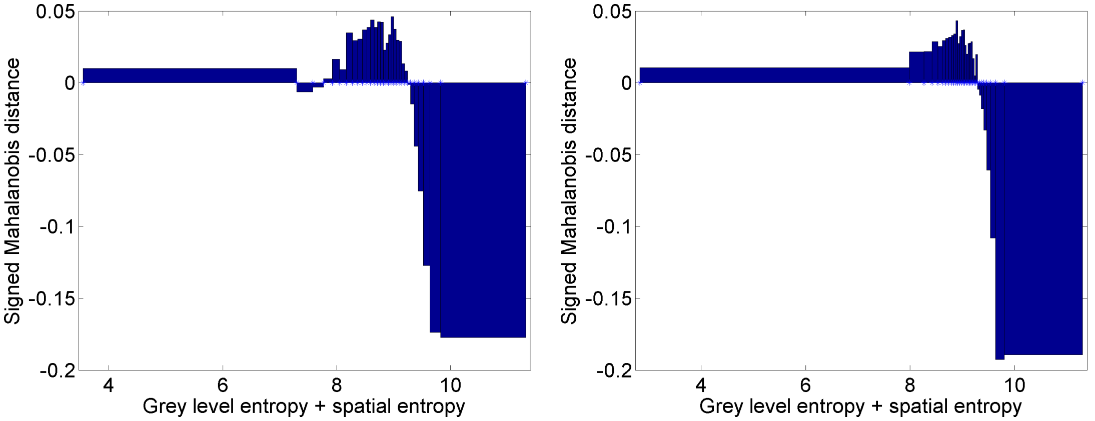

(a) **(b)**

Supplementary Figure S11. DESH weight vector for the (a) dark and (b) bright label when targeting 12000 entropy sums in each element. The weights were estimated using the nuclear images in the train subset of the endometrial carcinoma cohort.
DESH, dual entropy sum histogram.
